# Transcriptomic insights into early responses of the uterovaginal junction and vagina to avian influenza virus infection in turkey breeder hens

**DOI:** 10.3389/fphys.2025.1704399

**Published:** 2026-01-08

**Authors:** Sunantha Kosonsiriluk, Pitchaya Santativongchai, Kent M. Reed, Marissa M. Studniski, Ben W. Wileman, Kahina S. Boukherroub

**Affiliations:** 1 Department of Animal Science, University of Minnesota, Saint Paul, MN, United States; 2 Department of Veterinary and Biomedical Sciences, University of Minnesota, Saint Paul, MN, United States; 3 Select Genetics, Willmar, MN, United States

**Keywords:** avian influenza virus, low pathogenic avian influenza virus, transcriptomics, turkey, uterovaginal junction, vagina

## Abstract

**Introduction:**

Avian influenza virus (AIV) infections, even with low-pathogenic strains (LPAIVs), can severely disrupt reproduction in turkey breeder hens. Although the vagina and uterovaginal junction (UVJ) are among the earliest mucosal sites exposed to pathogens, their early transcriptomic responses to LPAIV infection are uncharacterized.

**Methods:**

This study investigated early transcriptomic changes in these tissues during both presymptomatic and symptomatic stages of LPAIV infection (n = 4/group/tissue). Flocks for sampling were classified as presymptomatic or symptomatic based on drinker swab LPAIV testing and egg production records. Presymptomatic group consisted of infected hens from LPAIV-negative barns. These flocks had stable egg production at the time of collection but with a subsequent egg drop. The symptomatic group included infected hens from LPAIV-positive barns with reduced egg production and sampled 2–3 days post-detection.

**Results:**

Principal component analysis of high-throughput RNA-seq data, identified symptomatic status as the primary driver of gene expression variance, followed by tissue origin. In the UVJ, 4,683 genes were differentially expressed (adjusted P-value < 0.05; Log2fold change ≥ 1.5), with symptomatic birds showing upregulation of genes involved in cellular remodeling and transport, and downregulation of those related to protein synthesis and metabolic pathways. Gene Set Enrichment Analysis (GSEA) confirmed significant upregulation of the insulin signaling pathway and downregulation of cytokine-cytokine receptor interaction, ribosome, and peroxisome proliferator-activated receptor signaling, indicating metabolic disruption and immune system alteration. The vagina presented a distinct transcriptomic profile, with 701 differentially expressed genes identified between the symptomatic vs. presymptomatic groups. In the symptomatic vagina, upregulated pathways were linked to gene regulation and biosynthesis, while downregulated pathways involved protein synthesis, metabolism, energy production, and vascular development.

**Discussion:**

These findings reveal early, tissue-specific molecular vulnerabilities to LPAIV. The UVJ shows disruptions in cellular maintenance and metabolism, potentially impairing fertility, while the vaginal response suggests heightened early immune activation but later compromised barrier integrity. This study offers potential mechanistic insights into LPAIV-induced reproductive pathologies, providing a foundation for targeted strategies to reduce viral impact on flock health and maintain production efficiency.

## 1 Introduction

Turkey breeder hens are particularly vulnerable to avian influenza virus (AIV) infections, posing significant challenges to flock health and production efficiency. During active egg production, hens experience increased physiological stress and elevated levels of reproductive steroid hormones, that transiently suppress immune function (Chen et al., 2024). This immunosuppressed state, combined with repeated mechanical and potentially microscopic mucosal injuries from artificial insemination (AI), creates favorable conditions for viral entry and replication (Kosonsiriluk et al., 2023). Repeated AI is a standard management practice in commercial turkey breeder operations, typically conducted weekly to enhance fertility (Artificial Insemination in Turkeys and Chickens - Poultry, 2025). However, this frequent handling increases the risk of AIV transmission, particularly via contaminated semen or equipment used during AI process.

Low-pathogenic avian influenza viruses (LPAIVs) are widely prevalent in commercial poultry, including turkeys, and represent a persistent challenge to poultry health and productivity (Criado et al., 2021). While LPAIVs are typically associated with subclinical or mildly symptomatic infections, they can nonetheless result in significant reproductive disturbances, including reduced egg production, compromised shell quality, and increased frequency of abnormal (e.g., soft- or thin-shelled) eggs (Swayne and Sims, 2021). These abnormalities may directly result from viral infection of reproductive tissues that interferes with normal oviduct and ovarian function (Stephens et al., 2020), and these effects can reduce the number of viable eggs and poults. In commercial settings, sudden declines in egg production have been documented during breeder flock outbreaks. Furthermore, LPAIV infection can decrease fertility and hatchability, likely due to direct and indirect effects on sperm storage, maintenance, and fertilization (Criado et al., 2021).

While the respiratory and gastrointestinal tracts are the primary natural routes of AIV infection (Pantin-Jackwood et al., 2017), cloacal and intrauterine pathways represent important alternative routes under intensive reproductive management. Notably, AIVs preferentially bind to glycan receptors containing sialic acid linked to galactose via an α2,3-glycosidic bond (SAα2,3Gal), that are abundantly expressed along the oviduct epithelium of turkey hens (Santativongchai et al., 2024). LPAIV infection in reproductive tissues such as the uterovaginal junction (UVJ) and vagina is of particular concern due to the functional significance of these sites in fertility and mucosal immunity. The UVJ contains specialized sperm storage tubules (SSTs) that enable prolonged storage of viable sperm, a critical mechanism supporting ongoing fertility between inseminations (Bobr et al., 1964; Freedman et al., 2001; Brady et al., 2023). The vagina serves as both a key barrier against ascending infections and an initial site of sperm selection, contributing to the elimination of pathogens and non-viable sperm before sperm reach the SSTs (Khillare et al., 2018).

While most AIV research has focused on systemic or respiratory tissues (Post et al., 2012; Ranaware et al., 2016; Dey et al., 2023), reproductive mucosal compartments remain largely understudied, despite their critical physiological and epidemiological roles. Although LPAIV is increasingly recognized for its detrimental effects on poultry reproduction, the early molecular events occurring in reproductive tissues prior to the onset of symptoms are not well characterized. The UVJ and vagina are key reproductive sites involved not only in fertility but also in local immune defense. Understanding how LPAIV affects gene expression in these tissues during the presymptomatic phase, is essential to uncovering the mechanisms of mucosal immune disruption and fertility loss. In this study, we compare gene expression profiles in the UVJ and vagina of turkey breeder hens during presymptomatic and symptomatic stages of LPAIV infection to identify early molecular alterations that may predict or contribute to disease progression and reproductive impairment. These findings will advance our understanding of avian reproductive mucosal immunity and inform future strategies to safeguard reproductive performance and enhance disease resilience in commercial turkey production systems.

## 2 Materials and methods

### 2.1 Experimental animals

Turkey breeder hens (*Meleagris gallopavo*) were obtained from a collaborating turkey breeding company. Hens were housed under standard commercial conditions (14.5-h light/9.5-h dark cycle) with a conventional 18% protein diet and water available *ad libitum*. At the time of sampling, hens were approximately 37 weeks old and in their sixth week of lay. Twenty hens were randomly selected from a parent stock breeder flock: ten from a barn with confirmed H1N2 AIV infection and ten from an adjacent barn showing no signs of egg production decline at the time of collection. Samples were categorized as presymptomatic or symptomatic at the barn level based on LPAIV detection status and egg production data. Presymptomatic samples were collected from a barn that had tested negative for LPAIV via drinker biofilm and tracheal swabs during routine surveillance in the week of sampling, when egg production was stable at approximately 75%. A subsequent egg production drop to below 10% coincided with a positive LPAIV test. Although all flock-level and bird-level surveillance tests during the sampling week were negative, retrospective diagnostic testing of harvested tissues revealed that viral RNA was already present in the reproductive tract, indicating that infection had begun prior to detectable changes in standard surveillance. Symptomatic samples were collected from a confirmed LPAIV-positive barn (drinker biofilm and tracheal swabs) approximately 2–3 days after detection, at which time egg production had also dropped to below 10%.

Of the 20 hens sampled, eight (four presymptomatic and four symptomatic) met predefined inclusion criteria for transcriptomic analysis. Birds were retained only if they had intact, matched UVJ and vaginal tissues, were in comparable active-lay physiological condition, and yielded RNA of sufficient integrity for sequencing. Samples were excluded when reproductive segments were missing or damaged, when hens showed signs of oviductal regression inconsistent with active lay, when RNA quality was inadequate, or when infection classification across tissues was inconsistent. These criteria were applied to avoid confounding and ensure biological comparability between groups.

The animal use protocol used in this study was approved by the University of Minnesota Institutional Animal Care and Use Committee (IACUC protocol no. 2204–39918A).

### 2.2 UVJ and vaginal tissue collection

Reproductive tracts from the twenty hens were removed and tissues dissected from the uterus to the vaginal opening. UVJ and vaginal tissues were separated, snap-frozen in liquid nitrogen, and stored at −80 °C until further processing.

### 2.3 RNA extraction and quantitative reverse transcription polymerase chain reaction (qRT-PCR)

Total RNA was extracted from UVJ and vaginal tissues using RNAzol® RT (MilliporeSigma, Burlington, MA, USA) according to the manufacturer’s instructions. RNA concentration and purity were assessed using a NanoDrop spectrophotometer, and integrity was verified using TapeStation 4200 (Agilent, Santa Clara, CA, USA).

Detection of AIV RNA was performed using the VetMAX™-Gold AIV Detection Kit (Thermo Fisher Scientific, Waltham, MA, USA) according to the manufacturer’s instructions. Real-time RT-PCR was conducted using the QuantStudio™ 3 Real-Time PCR System (Thermo Fisher Scientific, Waltham, MA, USA), with both positive and negative controls included in each run. Cycle threshold (Ct) values were recorded and interpreted as: Ct < 38, AIV-positive; Ct = 38-40, suspected AIV-positive; and non-detectable Ct, AIV-negative.

### 2.4 Statistical analysis

Cycle threshold values obtained from qRT-PCR were analyzed to assess differences in AIV RNA levels between symptomatic and presymptomatic groups. A two-way analysis of variance (ANOVA), followed by Tukey’s multiple comparison, was conducted to evaluate the effects of infection status (symptomatic vs. presymptomatic) and tissue type (UVJ vs. vagina) on Ct values. Where appropriate, unpaired Student’s t-tests were used for direct comparisons between two groups. A p-value less than 0.05 (p < 0.05) was considered statistically significant. All statistical analyses were performed using GraphPad Prism 9.

### 2.5 cDNA library construction and sequencing

Sixteen confirmed LPAIV-infected UVJ and vaginal samples (four birds per group across two tissue types and two groups: presymptomatic and symptomatic) were submitted to the University of Minnesota Genomics Center for RNA quantity and quality assessment using the RiboGreen assay (Thermo Fisher Scientific, Waltham, MA, USA) and TapeStation 4200 (Agilent, Santa Clara, CA, USA), respectively. Samples with RNA integrity numbers (RIN) ranging from 8.0 to 9.4 were used for cDNA library construction. Indexed libraries were constructed with the TruSeq Stranded mRNA Sample Preparation Kit (Illumina, Inc., San Diego, CA, USA) and size-selected for approximately 200 bp inserts. Libraries were multiplexed and sequenced on an Illumina NovaSeq 6,000 platform using a 150 paired-end flow cell to generate 51-bp paired-end reads.

### 2.6 Transcriptomic data analysis

RNA-Seq raw count data were initially processed using the integrated Differential Expression analysis Pipeline (iDEP v2.01), a web-based platform for differential expression and pathway analysis (https://bioinformatics.sdstate.edu/idep/) (Ge et al., 2018). Low-expression genes were filtered using iDEP’s default criterion, retaining genes with counts per million (CPM) ≥ 0.5 in at least one sample Expression values were transformed to log_2_(CPM +4) for visualization and exploratory analyses. Reads were aligned to the *M. gallopavo* Turkey_5.1 reference genome (ENSEMBL Taxonomy ID 9103).

For unsupervised analyses, log_2_(CPM +4)–transformed values were used for principal component analysis (PCA) and k-means clustering. K-means clustering was performed on the 2,000 most variable genes across samples to identify major transcriptional patterns. The number of clusters (k = 2) was selected to reflect the two major biological states under study (presymptomatic and symptomatic) and to provide a simplified representation of the global expression structure observed in the PCA and hierarchical clustering.

Differential expression analysis was performed using DESeq2 (Love et al., 2014), which applies its own internal normalization procedure. All statistical inferences, fold-change estimates, and FDR values were based exclusively on DESeq2-normalized counts. Pairwise comparisons between presymptomatic and symptomatic birds were performed within each tissue type. Genes with false discovery rate (FDR) < 0.05 and |log_2_ fold change| > 1.5 were considered differentially expressed.

To gain biological insights, functional enrichment analysis was performed on DEGs from each pairwise comparison to identify overrepresented Gene Ontology (GO) terms and Kyoto Encyclopedia of Genes and Genomes (KEGG) pathways (Ashburner et al., 2000; Kanehisa and Goto, 2000; Gene et al., 2023; Kanehisa et al., 2025). GO enrichment results were reviewed to reduce redundancy among hierarchical terms. Redundant or highly overlapping GO categories were consolidated under representative parent biological processes, and truncated GO terms were corrected for clarity. Gene Set Enrichment Analysis (GSEA) (Jambusaria et al., 2018) was applied to explore broader pathway-level changes, and Pathview (Luo and Brouwer, 2013) was used to map gene expression changes onto KEGG pathway diagrams.

All sequencing data are deposited in the NCBI Sequence Read Archive (SRA) under BioProject: PRJNA1305424.

## 3 Results

### 3.1 Confirmation of local LPAIV infection in the UVJ and vagina

To confirm active LPAIV infection and assess its presence in the reproductive tissues, viral RNA detection was performed using qRT-PCR detection kit targeting the AIV M gene. The AIV RNA was successfully detected in both the UVJ and vaginal tissues of all birds in both symptomatic and presymptomatic groups.

In symptomatic birds, AIV Ct values averaged 27.53 ± 2.37 in UVJ tissues (range: 25.12–30.08) and 31.17 ± 4.06 in vaginal tissues (range: 28.08–37.13). In presymptomatic birds, AIV Ct values averaged 31.96 ± 2.17 in UVJ tissues (range: 29.49–34.59) and 31.71 ± 2.69 in vaginal tissues (range: 28.80–35.04). Statistical analysis (t-tests) revealed a significant difference in AIV RNA levels between symptomatic and presymptomatic birds in UVJ tissues (p = 0.0009), but not in vaginal tissues (p = 0.830).

### 3.2 Gene expression profiles cluster according to symptomatic status

High-throughput RNA sequencing of the 16 samples produced a total of 525 million paired-end reads, averaging 30.9 million reads per sample, with quality scores (Q) ranging from 35 to 39. From these reads, 17,970 genes were detected as expressed across all samples, with 14,632 genes passing filtering criteria for downstream analysis.

**FIGURE 1:**
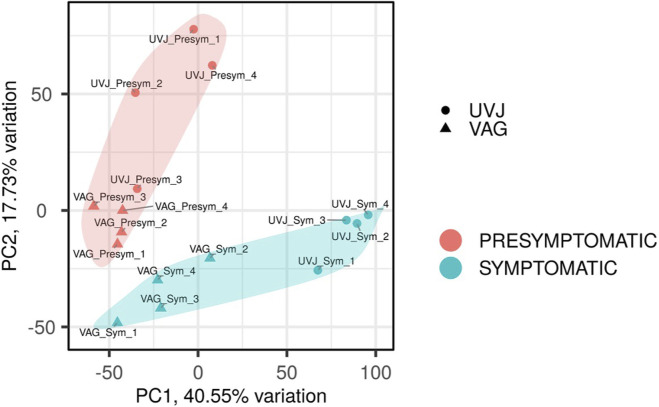
Principal component analysis (PCA) of transcriptomic profiles in turkey reproductive tissues during early low-pathogenic avian influenza virus (LPAIV) Infection. PCA of RNA-seq data from uterovaginal junction (UVJ, circles) and vaginal (VAG, triangles) tissues reveals global gene expression variation in presymptomatic (red shaded area) and symptomatic (cyan shaded area) LPAIV-infected turkeys. PC1 explains 40.55% of the variance and primarily separates samples by symptomatic status. PC2 accounts for 17.73% of the variance and contributes to within-group variation, especially among presymptomatic samples. The distinct clustering highlights the strong influence of symptomatic status on tissue-specific transcriptomic responses.

The PCA plot (Figure 1) revealed a clear clustering of samples, primarily driven by symptomatic status. PC1, accounting for 40.55% of the total variance, largely segregated samples based on symptomatic status. Presymptomatic samples predominantly clustered with negative PC1 values, while symptomatic UVJ samples grouped with positive PC1 values. Notably, symptomatic vaginal samples also clustered on the negative side of PC1, forming a distinct group from the symptomatic UVJ samples. While strong separation was observed, variability existed within clusters as exemplified by UVJ Presymptomatic sample 3, that was separated from the core presymptomatic cluster. An additional 17.73% of the variation was explained by PC2, which showed a discernible separation by sample origin. Specifically, UVJ samples generally exhibited higher PC2 values compared to VAG samples, particularly within the presymptomatic group.

### 3.3 Impact of LPAIV infection on transcriptional signatures in turkey reproductive tissues

**FIGURE 2:**
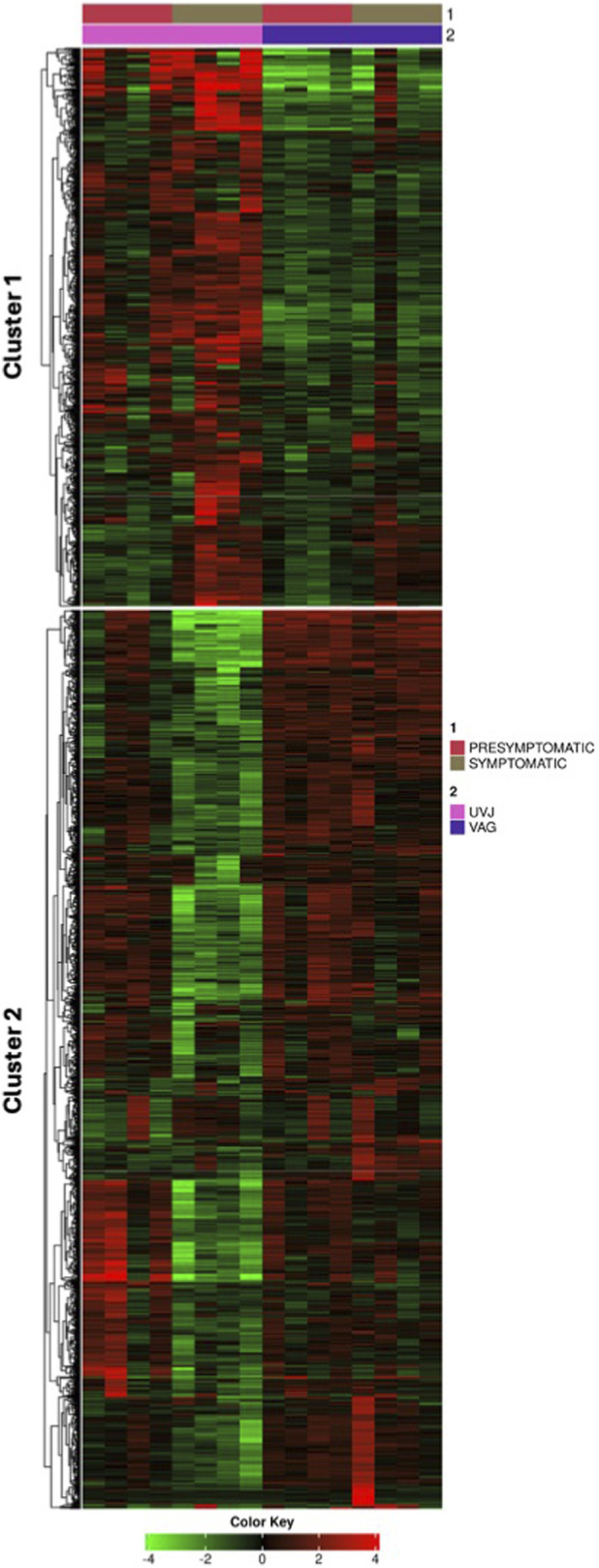
Global K-means clustering of gene expression profiles in turkey reproductive tissues. Heatmap showing K-means clustering of the 2,000 most variable genes across 16 uterovaginal junction (UVJ) and vaginal (VAG) tissue samples. Clustering was performed using log_2_(CPM +4)–transformed expression values. The top annotation bar (1) indicates symptomatic status (red = presymptomatic, olive green = symptomatic), and the second bar (2) indicates tissue type (pink = UVJ, purple = VAG). Hierarchical clustering of samples (columns) reveals strong grouping by symptomatic status, as shown in the dendrogram. Genes (rows) are clustered into two major expression groups: Cluster one is upregulated in symptomatic samples, while Cluster two is upregulated in presymptomatic samples. Gene expression is color-coded relative to the mean (green = downregulation, red = upregulation). The analysis highlights that symptomatic status is the primary driver of transcriptomic variation, with additional contributions from tissue type.

K-means clustering was performed on the 2,000 most variable genes across the 16 samples (Supplementary Table S1). As visualized in the heatmap (Figure 2), hierarchical clustering of strongly grouped samples according to infection stages, reinforcing the primary separation observed in the PCA. Within the genes, two major clusters (labeled Cluster one and Cluster two on the left) were evident. Gene cluster one exhibited a general pattern of upregulation (red) in symptomatic samples and downregulation (green) in presymptomatic samples. Gene cluster two largely showed downregulation in symptomatic samples and upregulation in presymptomatic samples.

Subsequent functional enrichment analysis of the highly variable genes that define these major expression patterns, revealed significant enrichment of several core biological pathways. Within gene cluster 1 (genes upregulated in symptomatic samples), highly enriched pathways included *ion transport* (adjusted FDR = 9.60 × 10^−11^, fold enrichment = 2.34) and *transmembrane transport* (adjusted FDR = 1.11 × 10^−7^, fold enrichment = 2.02). Conversely, within gene cluster 2 (genes downregulated in symptomatic samples), pathways such as *response to stimulus* (adjusted FDR = 3.83 × 10^−12^, fold enrichment = 1.38) and *immune response* (adjusted FDR = 2.30 × 10^−10^, fold enrichment = 2.36) were significantly enriched.

### 3.4 Immune and metabolic shifts in the UVJ during LPAIV infection

**FIGURE 3:**
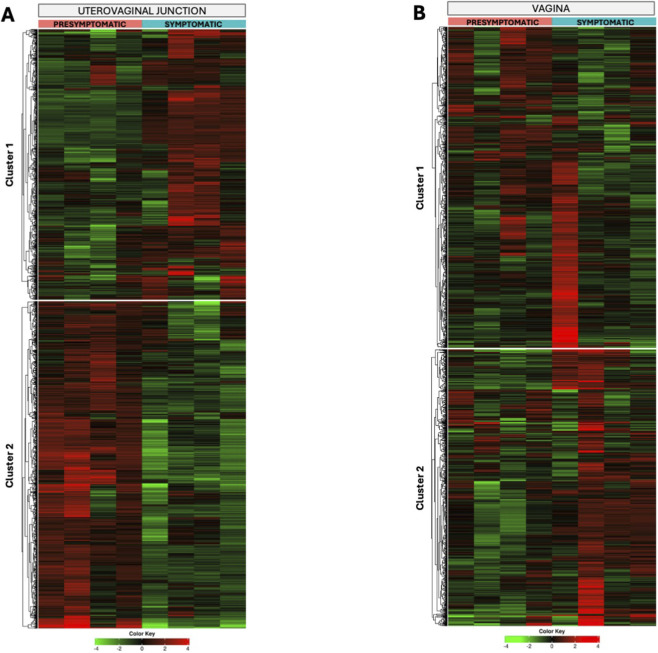
K-Means clustering of highly variable genes in the UVJ and vaginal tissues during LPAIV infection. Heatmap showing expression profiles of the 2,000 most variable genes in UVJ and vaginal tissues (**(A,B)**, respectively). For both tissues, clustering was performed using log_2_(CPM +4)–transformed values, and gene expression values were centered and scaled by row. Samples are primarily separated by symptomatic status (presymptomatic and symptomatic). Two major gene clusters are observed: Cluster one and Cluster 2. Columns represent individual turkey samples, color-coded by infection status (red = presymptomatic, cyan = symptomatic), and rows represent individual genes clustered by expression similarity. Gene expression is scaled relative to the mean, with green indicating downregulation and red indicating upregulation.

K-means clustering was performed on the 2,000 most variable genes to identify major sources of gene expression variability within the UVJ during LPAIV infection. The resulting heatmap (Figure 3A) revealed a clear hierarchical clustering of samples primarily by stages of infection, with presymptomatic and symptomatic UVJ samples forming distinct groups. Within the genes, two major clusters were identified; Gene Cluster 1 (Figure 3A) consisting of genes upregulated in symptomatic UVJ samples and downregulated in presymptomatic UVJ samples, and Gene Cluster 2 (Figure 3A) representing genes downregulated in symptomatic UVJ and upregulated in presymptomatic UVJ samples.

Functional enrichment analysis (KEGG Pathways) of Gene Cluster 2, (genes downregulated in symptomatic UVJ), showed significant enrichment in pathways predominantly related to immune signaling and cellular interactions. Key enriched pathways (FDR ≤0.0219, fold enrichment 2.36–3.80) included *cytokine-cytokine receptor interaction*, *ECM-receptor interaction*, *calcium signaling pathway*, and *phagosome*.

### 3.5 Transcriptional reprogramming in the UVJ during symptomatic LPAIV infection

**FIGURE 4:**
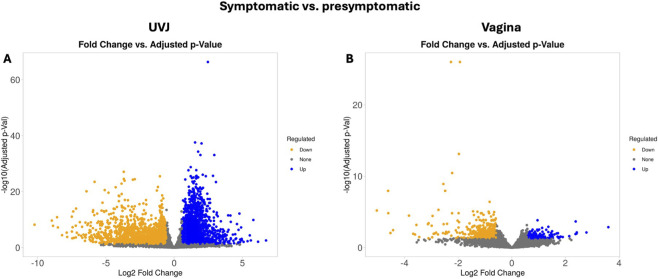
Volcano plot of differentially expressed genes in the UVJ and vagina during LPAIV infection (symptomatic vs. presymptomatic). Volcano plot showing the magnitude and significance of differential gene expression in the UVJ and vagina (**(A,B)**, respectively) between symptomatic and presymptomatic turkeys. The x-axis represents log_2_ fold change, with positive values indicating upregulation and negative values indicating downregulation in symptomatic samples. The y-axis shows–log_10_ of the adjusted p-value, with higher values indicating greater statistical significance. Genes are color-coded by significance and direction: orange = significantly downregulated, blue = significantly upregulated, and grey = not significantly differentially expressed.

To identify specific molecular alterations occurring in the UVJ as LPAIV infection progresses, we compared gene expression in the UVJ tissues of symptomatic vs. presymptomatic turkey hens. Using DESeq2, we identified a total of 4,682 DEGs (FDR <0.05 and Log_2_FC > 1.5). Of these, 2,519 were significantly upregulated in symptomatic UVJ samples compared to presymptomatic UVJ samples, and 2,163 genes were significantly downregulated in symptomatic UVJ (Supplementary Table S2). Expression values reflect the log-transformed CPM scale used in iDEP. A volcano plot (Figure 4A) visually highlights these extensive transcriptional changes in the UVJ, showing numerous genes exceeding the significance and fold change thresholds.

Functional enrichment analysis (Gene Ontology Biological Process) of these 4,683 DEGs revealed a distinct array of biological pathways significantly altered in symptomatic UVJ compared to presymptomatic UVJ (Table 1). Pathways significantly upregulated in symptomatic UVJ were prominently associated with cellular modification and transport processes (e.g., *cellular protein modification process, transport, localization, macromolecule modification, organic substance transport,* and *ion transport*), and intracellular signaling (e.g., *intracellular signal transduction* and *phosphorylation*). Conversely, pathways significantly downregulated in symptomatic UVJ primarily involved fundamental cellular processes such as protein synthesis and metabolism (e.g., *translation, peptide biosynthetic process, amide biosynthetic process,* and *ribosome biogenesis*) as well as cellular organization and communication (e.g., *cell adhesion* and *chemotaxis*).

**TABLE 1:** Representative enriched gene ontology (GO) biological process pathways for differentially expressed genes in the UVJ, comparing symptomatic to presymptomatic turkeys.

Direction	Adj.Pval	# Genes	Fold enrichment	Pathway
Upregulated	1.93E-06	399	1.28	Cellular protein modification process
	1.93E-06	431	1.28	Transport
	1.93E-06	539	1.22	Localization
	1.25E-02	219	1.28	Intracellular signal transduction
	1.95E-02	200	1.29	Phosphorylation
	2.05E-02	153	1.34	Ion transport
Downregulated	5.7E-10	102	2.05	Translation
	8.85E-10	103	2.01	Peptide biosynthetic process
	1.6E-08	111	1.85	Peptide metabolic process
	4.11E-06	119	1.66	Cellular amide metabolic process
	2.28E-05	106	1.67	Cell adhesion
	4.1E-05	43	2.29	Ribosome biogenesis
	7.81E-05	54	2.03	Ribonucleoprotein complex biogenesis
	6.46E-04	126	1.49	Cellular macromolecule biosynthetic process
	5.53E-03	48	1.84	Chemotaxis

### 3.6 Pathway enrichment reveals coordinated shifts in UVJ signaling and metabolism

To further identify subtle but coordinated pathway-level changes not fully captured by differential expression thresholds, GSEA was performed on UVJ samples comparing symptomatic vs. presymptomatic birds, using the KEGG database. The full list of significantly enriched pathways (adjusted P-value <0.05), along with their Normalized Enrichment Scores (NES) and adjusted P-values, is presented in Table 2.

**TABLE 2:** KEGG pathways enrichment in the UVJ using GSEA, comparing symptomatic to presymptomatic turkeys.

Direction	Pathway	NES	# Genes	Adj.Pval
Up	Insulin signaling pathway	0.5204	45	2.2E-02
Down	Ribosome	−0.6925	46	1.3E-04
	Cytokine-cytokine receptor interaction	−0.6139	76	2.2E-04
	Neuroactive ligand-receptor interaction	−0.5333	94	4.1E-03
	PPAR signaling pathway	−0.6692	28	1.5E-02
	Vascular smooth muscle contraction	−0.6058	43	1.9E-02
	Phagosome	−0.5628	53	2.2E-02

NES, normalized enrichment score. Pathways with FDR-adjusted p-values <0.05 are reported.

The upregulated pathways in symptomatic UVJ were notably enriched for *insulin signaling pathway*. Among pathways downregulated in symptomatic UVJ samples, prominent processes included *ribosome, cytokine-cytokine receptor interaction, neuroactive ligand-receptor interaction, Peroxisome proliferator-activated receptors (PPAR) signaling pathway, vascular smooth muscle contraction*, and *phagosome*.

**FIGURE 5:**
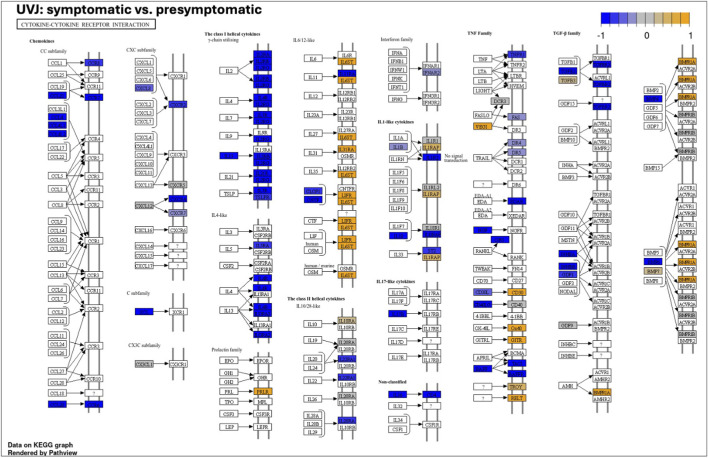
KEGG pathway map of cytokine–cytokine receptor interaction in the UVJ during LPAIV Infection. KEGG pathway map illustrating differential gene expression in the cytokine–cytokine receptor interaction pathway in UVJ tissues comparing symptomatic vs. presymptomatic turkeys. Each box represents a gene, with connecting lines indicating known interactions. Color coding reflects expression changes: orange = upregulated, blue = downregulated, and grey = no changes in expression. The color scale (top right) reflects relative expression (log_2_ fold change), ranging from −1 (blue) to +1 (yellow). This figure highlights the cytokine signaling network activated in the UVJ during early LPAIV infection, with a predominance of downregulated immune-related genes in symptomatic samples.

**FIGURE 6:**
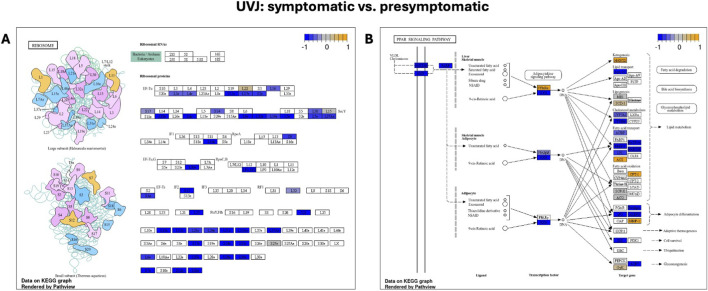
KEGG pathways downregulated in the UVJ during symptomatic LPAIV infection. **(A)** *Ribosome pathway* and **(B)** P*eroxisome proliferator-activated receptor (PPAR) signaling pathway* diagrams from KEGG illustrate pathway components in UVJ tissues comparing symptomatic to presymptomatic turkeys. Each box represents a gene, with color indicating differential expression: orange = upregulated, blue = downregulated, and grey = no changes in expression. The color scale (top right) reflects relative expression (log_2_ fold change), ranging from −1 (blue) to +1 (yellow). These pathways show coordinated downregulation during symptomatic LPAIV infection, suggesting disruptions in protein synthesis and metabolic regulation.

A detailed visualization of the cytokine-cytokine receptor interaction pathway (Figure 5) illustrates that while the overall pathway exhibited strong negative enrichment (NES: 0.6139) indicating a general downregulation of its components, there was a nuanced response. Specifically, genes such as *CXCL8*, *IL-15*, *IL2R*, *IL4R*, *IL7R*, and *IL21R* exhibited distinct expression patterns within this pathway across samples. As depicted in Figure 5, while the majority of components were downregulated (colored blue), these and other individual genes showed varying expression, including upregulation (colored yellow), collectively driving the significant negative enrichment score. Similarly, detailed visualizations for the *ribosome* (Figure 6A) and *PPAR signaling* pathways (Figure 6B) showed overall downregulation (colored blue) in symptomatic UVJ samples, consistent with their negative enrichment.

### 3.7 Distinct immune, adhesion, and calcium signaling patterns in the vagina during LPAIV infection

To complement the UVJ analysis and investigate the major sources of gene expression variability specifically within the vagina, K-means clustering was performed on the 2,000 most variable genes exclusively from vaginal samples. The resulting heatmap (Figure 3B) revealed a clear clustering of samples primarily by stages of infection, consistent with observations in the UVJ and overall dataset, with presymptomatic vaginal samples grouping distinctly from symptomatic vaginal samples. Two major gene clusters were identified; Gene Cluster 1 (Figure 3B) generally exhibiting downregulation (green) in symptomatic samples and upregulation (red) in presymptomatic samples, and Gene Cluster 2 (Figure 3B) showing upregulation (red) in symptomatic samples and downregulation (green) in presymptomatic samples.

Functional enrichment analysis (KEGG Pathways) found Gene Cluster 1, comprising genes downregulated in symptomatic vagina, significantly enriched in pathways related to immune signaling and cellular adhesion. Specific pathways included *cytokine-cytokine receptor interaction* (adjusted P-value = 5.73 × 10^−5^, Log_2_fold change = 3.1) and *cell adhesion molecules* (adjusted P-value = 3.35 × 10^−3^, Log_2_fold change = 3.5). Gene Cluster 2, containing genes upregulated in symptomatic vagina, was significantly enriched in the *calcium signaling pathway* (adjusted P-value = 2.67 × 10^−2^, Log_2_fold change = 2.9). This suggests that changes in cellular signaling pathways related to calcium homeostasis constitute a notable expression shift in the vagina during symptomatic infection.

### 3.8 Transcriptional reprogramming in the vagina during symptomatic LPAIV infection

Gene expression changes (FDR <0.05 and Log_2_FC > 1.5) in the vaginal tissue are summarized in Figure 4B where 701 DEGs were identified. Of these, 554 genes were significantly upregulated (blue dots) in symptomatic vaginal samples compared to presymptomatic vaginal samples, while 147 genes were significantly downregulated (orange dots) in symptomatic vagina (Supplementary Table S2).

**FIGURE 7:**
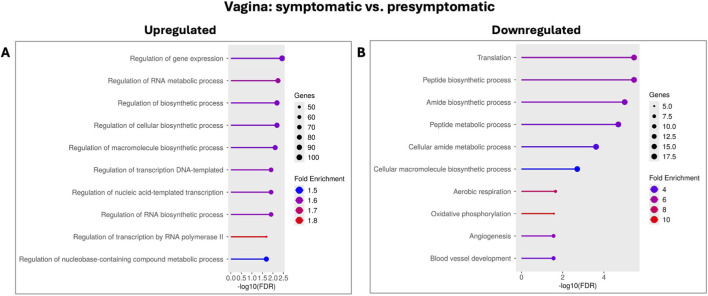
Gene Ontology (GO) enrichment of differentially expressed genes in the vagina during LPAIV infection (symptomatic vs. presymptomatic). GO biological process enrichment analysis comparing symptomatic to presymptomatic vaginal samples. **(A)** Upregulated pathways: Top enriched GO terms among genes upregulated in symptomatic samples. **(B)** Downregulated pathways: Top enriched GO terms among genes downregulated in symptomatic samples. These results highlight key biological processes altered during early LPAIV infection in vaginal tissue.

Functional enrichment analysis (Gene Ontology Biological Process) of these 701 DEGs in the vagina revealed a distinct array of biological pathways significantly altered in symptomatic vaginal samples compared to presymptomatic vaginal samples (Table 3). Pathways significantly upregulated in symptomatic vagina were associated with regulation of gene expression and various biosynthetic processes (e.g., *regulation of gene expression, regulation of RNA metabolic process, regulation of biosynthetic process, regulation of cellular biosynthetic process,* and *regulation* *of macromolecule biosynthetic process*, Figure 7A). Conversely, pathways significantly downregulated in symptomatic vagina primarily involved fundamental cellular processes such as protein synthesis and metabolism, energy production, and vascular development (e.g., *translation, peptide biosynthetic process, amide biosynthetic process, aerobic respiration, oxidative phosphorylation, angiogenesis*, and *blood vessel development*, Figure 7B).

**TABLE 3:** Representative enriched gene ontology (GO) biological process pathways for differentially expressed genes in the vagina, comparing symptomatic to presymptomatic turkeys.

Direction	Adj.Pval	# Genes	Fold enrichment	Pathway
Upregulated	3.62E-03	101	1.56	Regulation of gene expression
	5.71E-03	82	1.62	Regulation of RNA metabolic process
	1.23E-02	73	1.59	Regulation of transcription DNA-templated
	2.06E-02	45	1.81	Regulation of transcription by RNA polymerase II
Downregulated	3.45E-06	18	5.51	Translation
	3.45E-06	18	5.35	Peptide biosynthetic process
	2E-05	18	4.57	Peptide metabolic process
	2.4E-04	18	3.81	Cellular amide metabolic process
	1.97E-03	18	3.25	Cellular macromolecule biosynthetic process
	2.23E-02	6	8.10	Aerobic respiration
	2.68E-02	5	10.13	Oxidative phosphorylation
	2.78E-02	8	5.13	Angiogenesis
	2.78E-02	9	4.26	Blood vessel development

**FIGURE 8:**
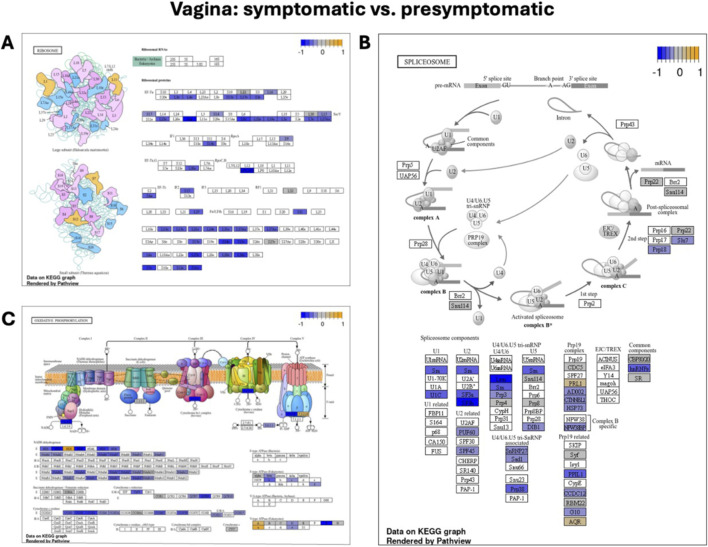
KEGG pathways downregulated in the vagina during symptomatic LPAIV infection. **(A)** *Ribosome*, **(B)** *Oxidative phosphorylation*, and **(C)** *Spliceosome* pathway diagrams from KEGG illustrate pathway components in vaginal tissues comparing symptomatic to presymptomatic turkeys. Each box represents a gene, with color indicating differential expression: orange = upregulated, blue = downregulated, and grey = no changes in expression. The color scale (top right) reflects relative expression (log_2_ fold change), ranging from −1 (blue) to +1 (yellow). These pathways exhibit coordinated downregulation during symptomatic LPAIV infection, indicating reduced protein synthesis, mitochondrial function, and RNA processing in the vaginal tissue.

GSEA analysis revealed several significant downregulated pathways in symptomatic samples. Most notably, *ribosome* (NES = −0.81, adjusted P-value = 1.7e-15), *oxidative phosphorylation* (NES = −0.55, adjusted P-value = 4.3e-05), and *spliceosome* (NES = −0.51, adjusted P-value = 4.3e-05) pathways were significantly enriched (Figures 8A–C, respectively).

## 4 Discussion

### 4.1 Tissue-specific transcriptional responses during LPAIV infection

Initial confirmation of LPAIV RNA in both UVJ and vaginal tissues across all symptomatic and presymptomatic birds established the foundational context for our transcriptomic investigation. Symptomatic birds exhibited a decline in egg production and an increase in cull eggs (thin shells, white shells with little to no pigment, eggs laid with no shell, wrinkled shells, etc.). A significant difference in overall viral RNA load (as measured by Ct values) was observed in the UVJ, but not in vaginal tissues, between symptomatic and presymptomatic birds. This pattern may be influenced by the timing of sample collection that can affect viral detection in the UVJ. These findings suggest that the transcriptomic changes observed in the UVJ reflect both increased viral presence and the host’s dynamic response to infection, which differs from the more limited response observed in the vagina, highlighting the tissue-specific nature of transcriptional reprogramming during disease manifestation.

A limitation of this study is the absence of a traditional uninfected control group. Because sampling occurred during a naturally circulating LPAIV outbreak, hens classified as presymptomatic were initially considered negative based on flock-level drinker biofilm testing, random bird-level tracheal swabs, and stable egg production at the time of sampling. Retrospective diagnostics revealed that viral RNA was already present in reproductive tissues. Viral replication can begin during a period when both flock-level and bird-level surveillance remain negative and when clinical or production changes have not yet emerged, which makes obtaining truly uninfected birds extremely difficult under field conditions. Despite this constraint, the comparison between presymptomatic and symptomatic hens captures a very early stage of natural infection and provides meaningful insight into the initial molecular disruptions associated with LPAIV establishment.

### 4.2 Transcriptional dynamics in the UVJ and vagina in response to LPAIV infection

PCA (Figure 1) and global K-means clustering (Figure 2) found symptomatic LPAIV infection was the predominant driver of gene expression variation across the turkey reproductive tissues, accounting for over 40% of the variance. Although the UVJ and vagina are anatomically proximate, their distinct molecular responses to infection reflect the specialized functions of their respective tissues. Genes upregulated in symptomatic samples (Cluster 1, Figure 2) were enriched for *ion transport* and *transmembrane transport* pathways suggesting an attempt to restore basic cellular function following disruption by LPAIV infection. These transport pathways appeared to be more profoundly affected in the UVJ, potentially due to its unique role in sperm storage (Kosonsiriluk et al., 2023), where such disruptions could severely impair function. Although the vagina also showed some changes in these genes, their overall expression levels were generally lower compared to the UVJ, suggesting a more nuanced involvement of these specific transport mechanisms in the vaginal response. Genes in Cluster 2 (Figure 2) were enriched for pathways related to *stimulus response* and *immune activation*, with an expression pattern characterized by sustained upregulation in the vagina and transient upregulation in the presymptomatic UVJ. This suggests the vagina maintains a prolonged immune response throughout the progression from presymptomatic to symptomatic stages, while the UVJ shows a more dynamic pattern, initially increasing and then decreasing expression. Such a pattern may reflect the need for the UVJ to restore tissue homeostasis while limiting prolonged immune activation, which could be detrimental to its function as a sperm storage site.

### 4.3 Immune alterations in the UVJ during early LPAIV infection

In the UVJ, a key tissue for sperm storage and fertility, the observed molecular responses were consistent with disrupted local immune responses in the reproductive tissues. K-means clustering of highly variable genes (Figure 3A) identified a major cluster associated with immune signaling and cellular remodeling, including *cytokine–cytokine receptor interaction, phagosome formation, cell adhesion molecules (CAMs),* and *ECM-receptor interaction*. Among these pathways, *cytokine-cytokine receptor interaction* was significantly enriched in a downregulated direction in symptomatic vs. presymptomatic comparison. This pathway is fundamental to intercellular communication, particularly in the immune system, and includes interleukins, chemokines, tumor necrosis factors, colony-stimulating factors, activins, and interferons, along with their corresponding receptors (Aggarwal, 2003; Turner et al., 2014; Akdis et al., 2016). Enrichment of this pathway indicates an active and finely regulated immune microenvironment, where communication between immune cells and other stromal cells is continuous (Han et al., 2019). This dynamic interaction is essential for maintaining the delicate balance required for physiological functions and for responding to potential challenges, such as the presence of spermatozoa (Khillare et al., 2018; Han et al., 2019). This result further supports a temporal shift, with these immune genes heightened early in presymptomatic birds, followed by a decline in symptomatic birds, suggesting a transient immune activation that does not persist.

Chemokines, small cytokines functioning as chemoattractant, guide migration of immune cells to sites of inflammation and infection and play a crucial role in recruiting phagocytes (Sokol and Luster, 2015). As demonstrated in our GSEA analysis (Figure 5), expression patterns of genes such as *CXCL8, IL-15, IL2R, IL4R, IL7R,* and *IL21R* within the *cytokine-cytokine receptor interaction* pathway suggest an active inflammatory response occurring early, before the onset of symptoms in presymptomatic UVJ tissue (Hama et al., 2009; Jiang et al., 2013). These interleukin-related genes are primarily involved in the proliferation, differentiation, and survival of T lymphocytes, particularly cytotoxic T cells, that are critical for cell-mediated antiviral immunity (Tarpey et al., 2007; Hao et al., 2023). In avian species, IL-2 enhances T cell responses and reduces viral loads (Jiang et al., 2013). As infection progressed (indicated by drop in egg production and an increase in cull eggs), this pathway was broadly downregulated in symptomatic tissues, potentially reflecting immune suppression, exhaustion, or viral modulation of host signaling. While a subset of components remained upregulated, the predominance of downregulated genes suggests a transition toward a dysregulated or inadequate immune state, impairing the tissue’s ability to clear the virus or maintain homeostasis.

Reduced expression of key chemokines may limit the recruitment of immune cells to the UVJ, reducing the effectiveness of the host’s antiviral defense and delaying the resolution of inflammation. The presence of upregulated components amidst broader downregulation might reflect a localized attempt by the host to sustain immune responses or a viral strategy to manipulate specific immune functions. Although studies have reported IFN-λ and IL-2 upregulation in avian oviduct tissues following LPAIV infection (Wang et al., 2015), if inconsistently expressed across symptomatic birds or counterbalanced by stronger downregulation of other critical cytokines, the net outcome may still be immunosuppression. Broad dampening of cytokine-mediated communication could contribute to the pathology observed in symptomatic birds by disrupting the reproductive microenvironment essential for sperm viability.

These observations suggest a temporal progression of immune responses, with an initial proinflammatory activation phase in presymptomatic birds, followed by a collapse or suppression of cytokine signaling in the symptomatic phase. The local immune responses in the UVJ are crucial for defending against potential pathogens while simultaneously creating a tolerogenic environment for spermatozoa (Atikuzzaman et al., 2015). However, this delicate balance is susceptible to dysregulation, that can have significant consequences for sperm viability (Barratt and Pockley, 1998). This pattern may help explain the transition to symptoms and compromised fertility highlighting the importance of time-resolved immune profiling in understanding host–pathogen interactions in avian reproductive tissues.

### 4.4 LPAIV infection shifts structural and metabolic of the UVJ

Beyond immune signaling, phagosome formation also emerged as a significant biological process enriched in the UVJ gene clusters. Phagocytosis is a fundamental cellular mechanism by which cells engulf and degrade foreign particles, pathogens, or cellular debris, such as apoptotic bodies. This process is critical for host defense and tissue homeostasis, involving complex steps including actin polymerization, vesicle trafficking, and membrane remodeling (Han et al., 2019). In the UVJ, expression of genes associated with phagosome formation suggests an active role of phagocytic cells in immune surveillance and the removal of compromised cells or foreign entities. This continuous cleansing mechanism is crucial for maintaining a healthy environment with a low microbial burden, particularly important in a region like the UVJ where spermatozoa are stored and protected from excessive immune activation, while still defending against pathogens. The capacity for efficient phagosome formation ensures that the UVJ can effectively manage cellular waste and potential immunological challenges. The other enriched pathways include *CAMs* and *ECM-receptor interaction*. *CAMs* constitute a crucial category of proteins that facilitate cell-cell and cell-ECM interactions (Freemont and Hoyland, 1996; Skubitz, 2002). Beyond their structural role in adhesion, *CAMs* are integral to the inflammatory response and can modulate cytokine synthesis, highlighting their multifaceted roles in immune regulation (Han et al., 2024). In the UVJ, the enrichment of genes related to *CAMs* and *ECM-receptor interaction* highlights their importance in maintaining the structural integrity of the tissue, mediating cell migration, and regulating immune cell interactions within the local microenvironment.

Complementing this, other UVJ gene patterns indicated a coordinated suppression of core metabolic pathways in symptomatic UVJ tissue (Table 1) as confirmed by DEG analysis. Pathways involved in heightened protein synthesis (e.g., *translation* and *ribosome biogenesis*) appeared active early in the presymptomatic group, suggesting immune defense or viral replication, but were subsequently lowered once birds showed symptoms. A significant upregulation of pathways critical for cellular maintenance, such as protein modification, various transport processes, and intracellular signaling were enhanced later in symptomatic group compared to presymptomatic group suggesting a direct compromise of the UVJ’s intrinsic machinery once disease progresses. Further reinforcing these themes, the overall downregulation of *ribosome* and *phagosome* pathways, together with an upregulation of the *insulin signaling pathway* (Table 2), implies profound systematic metabolic alteration.

The *insulin signaling pathway* is central to regulating glucose homeostasis, energy metabolism, cell growth, and survival and was upregulated in symptomatic UVJ samples compared to presymptomatic ones. This upregulation suggests an active response to restore UVJ function, such as sperm sustenance, storage, and secretory activities, perhaps compensating for earlier metabolic suppression or addressing increased energy demands during symptomatic infection. Compromised insulin signaling early in infection (implied by its later upregulation) could lead to reduced nutrient availability for resident cells and stored sperm, potentially affecting sperm viability and fertility. This metabolic shift could be a consequence of the systemic stress associated with progressive infection, a direct effect of viral interference with host metabolic pathways to create an environment favorable for viral replication, or a host energy-conservation strategy that unfortunately compromises reproductive function (Arneth, 2023; Makhijani et al., 2023; Darweesh et al., 2025).

Studies on AIV infection in ducks have demonstrated significant enrichment for genes involved in ribosome function, protein metabolism, and gene expression in infected lung cells (Ndimukaga et al., 2020). This suggests that AIVs may interfere with host translation machinery, either as a viral strategy to evade host defenses or due to host-mediated suppression of viral protein synthesis prior to translation in the cytosol. Consistent with this, our analysis of the UVJ transcriptome revealed marked downregulation of pathways related to *ribosome structure*, *ribosome biogenesis*, and *translation* in symptomatic birds (Tables 1, 2; Figure 3A). While the role of *PPAR signaling* in LPAIV infection remains underexplored, emerging evidence points to potential crosstalk between ribosomal activity and PPAR-mediated metabolic regulation during viral infections. Both pathways respond to viral presence, and some studies suggest direct or indirect interactions (Gray et al., 2006; Ye et al., 2019). Together, these findings indicate that LPAIV infection in the UVJ disrupts essential cellular processes and metabolic homeostasis, impairing the tissue’s ability to support sperm storage and thereby compromising fertility.

### 4.5 LPAIV infection elicits rapid robust immune activation and calcium signaling pathway alteration in vaginal tissue

In parallel, the vagina, a critical immune barrier, exhibited a distinct molecular response to LPAIV infection. K-means clustering (Figure 3B) revealed infection status as the primary driver of transcriptional changes in vaginal tissue. Promptly upregulated genes in presymptomatic samples were significantly enriched in immune-related pathways, including *cytokine-cytokine receptor interaction* and *cell adhesion molecules*. This expression pattern reflects the vagina’s role as a frontline defense tissue, actively initiating an inflammatory response and regulating immune cell recruitment and epithelial integrity even before the onset of symptoms in LPAIV-infected birds (Abdel-Mageed et al., 2014; Wigley et al., 2022).

A particularly striking observation was the significant upregulation of the *calcium signaling pathway* in symptomatic vaginal tissues. Calcium signaling plays a central role in rapid cellular processes such as smooth muscle contraction (essential for egg transport), immune cell activation, and maintenance of epithelial tight junctions (Yano et al., 2010; Bleich et al., 2012; Mahapatra and Kumar, 2024). This upregulation in the symptomatic phase, following a potential suppression early in LPAIV infection, could signify a robust, albeit potentially dysregulated, attempt by the host to restore its physiological functions or combat the infection. This intense increase might be a compensatory mechanism to strengthen physical barrier function, enhance immune cell responses (e.g., increased calcium flux in T-cells or macrophages), or respond to tissue damage caused by LPAIV. Alternatively, it may reflect a viral strategy to manipulate host cellular processes, potentially affecting tissue integrity or muscular activity to facilitate viral egress or spread (Sid et al., 2017; Saurav et al., 2021; Anton et al., 2022). This distinct pattern of enhanced calcium signaling points towards a specific molecular response in the vagina, attempting to bolster immediate defense capabilities and physiological transit functions, possibly in an effort to restore to a normal functional level.

### 4.6 LPAIV modulation of host translational and metabolic pathway in vaginal tissue

Our analysis of symptomatic vaginal tissues revealed significant downregulation of several fundamental cellular pathways, including *ribosome function*, *oxidative phosphorylation*, and *spliceosome activity* (Figures 8A–C). This observation is consistent with general viral strategies, as many viral infections, particularly those caused by influenza A viruses, are known to disrupt a range of host cellular processes to their advantage.

Many viruses, for instance, initiate host protein synthesis shutoff, thereby redirecting cellular machinery to favor viral replication (Geiss et al., 2001; Kleinman et al., 2014; Wang et al., 2022). They can impair ribosome biogenesis, interfere with the assembly of mature ribosomes, and modulate mRNA recognition by the ribosomal complex. These strategies facilitate viral protein synthesis while suppressing the production of host antiviral proteins (Wang et al., 2022). Similarly, while some viruses upregulate oxidative phosphorylation to meet energy demands for replication, others have been shown to downregulate mitochondrial biogenesis and impair oxidative phosphorylation, leading to metabolic dysregulation (Dai et al., 2017; Guarnieri et al., 2022; Purandare et al., 2023). Influenza A virus has is reported to induce alternative splicing of host genes, promoting the expression of viral-supportive isoforms and further enhancing replication efficiency (Thompson et al., 2020). The downregulation of these critical pathways in LPAIV-infected vaginal tissue thus points to sophisticated strategies employed by the virus to hijack and manipulate host cell processes, ensuring its replication, persistence, and evasion of immune defenses within this reproductive site.

### 4.7 Molecular landscapes and viral dissemination in the turkey reproductive tract during LPAIV infection

A particularly insightful observation regarding viral dissemination was revealed by qRT-PCR analysis: in presymptomatic birds where viral RNA was readily detected in both the vagina and UVJ, yet conspicuously absent from the uterus (data not shown). This contrasts sharply with symptomatic birds, where viral RNA was consistently found across all three reproductive tissues, vagina, UVJ, and uterus (data not shown). This distinct pattern suggests a potential route and progression of infection: the vagina may serve as the initial point of introduction for LPAIV, from where it spreads locally to the adjacent UVJ, and subsequently ascends to the uterus as the infection progresses (Pantin-Jackwood et al., 2010).

Our study appears to have captured the infection at an early enough stage to discern this differential viral distribution, providing insights into a potential timeline where the vagina and UVJ become positive before the uterus. This sequential detection of viral RNA also suggests that a systemic spread from different entry routes simultaneously infecting all reproductive tissues is less likely. Given that the uterus is known to possess AIV receptors (Wang et al., 2015), if systemic dissemination were the primary mode of infection, one would expect all susceptible tissues to be concurrently infected. The observed progression, however, strongly supports a local, ascending spread within the reproductive tract. While the qPCR results for the uterus are not included in the scope of this study, this finding offers a compelling hypothesis for future investigation into the precise dynamics of LPAIV dissemination within the hen’s reproductive system.

In conclusion, our multi-layered transcriptomic analysis unequivocally reveals both commonalities and critical tissue-specific distinctions in the turkey reproductive tract’s response to LPAIV infection. While both the UVJ and vagina mount robust immune responses, their additional molecular perturbations diverge in ways consistent with their specialized roles. The UVJ demonstrates a profound disruption of metabolic and cellular maintenance pathways, impacting its sperm storage and fertility functions. In contrast, the vagina exhibits an intensified immune barrier response coupled with a unique impairment of calcium signaling, potentially compromising its integrity as a first-line defense and its role in egg passage.

Overall, transcriptional changes observed in presymptomatic birds suggest that molecular disruption begins well before symptoms are apparent. These early changes initiate damage that the reproductive tract subsequently attempts to repair or balance in order to restore homeostasis. This temporal insight is critical: we typically detect LPAIV infection only after birds exhibit overt symptoms or marked drops in egg production. However, if a robust screening system were implemented to catch infections in the presymptomatic phase and paired with preventative interventions, it could significantly reduce the impact of LPAIV on flock health, limit viral spread, and help maintain production efficiency.

This study identified specific molecular pathways as potential targets for future intervention. Modulating these pathways may minimize the detrimental impacts of infection and accelerate the restoration of reproductive function, thereby enhancing the vagina’s barrier defense and preserving the UVJ’s role in fertility maintenance. Together, the distinct molecular landscapes of the UVJ and vagina provide a strong mechanistic basis for the observed reproductive pathologies in infected turkeys, such as reduced egg production, altered egg quality, and compromised fertility. Understanding these tissue-specific vulnerabilities is crucial for developing targeted mitigation strategies. Future studies should aim to validate the key gene and protein alterations identified here, and explicitly link them to viral kinetics, histopathological outcomes, and reproductive performance metrics to further elucidate their roles in LPAIV pathogenesis.

## Data Availability

The datasets presented in this study can be found in online repositories. The names of the repository/repositories and accession number(s) can be found below: NCBI, BioProject, accession number PRJNA1305424.
